# Identification of a Twelve-Gene Signature and Establishment of a Prognostic Nomogram Predicting Overall Survival for Medulloblastoma

**DOI:** 10.3389/fgene.2020.563882

**Published:** 2020-09-03

**Authors:** Sihan Zhu, Fuhua Lin, Zhenghe Chen, Xiaobing Jiang, Ji Zhang, Qunying Yang, Yinsheng Chen, Jian Wang

**Affiliations:** Department of Neurosurgery and Neuro-Oncology, State Key Laboratory of Oncology in South China, Collaborative Innovation Center for Cancer Medicine, Sun Yat-sen University Cancer Center, Guangzhou, China

**Keywords:** medulloblastoma, nomogram, GEO, overall survival, gene signature

## Abstract

**Background:**

Medulloblastoma is the common pediatric malignant tumor with poor prognosis in cerebellum. However, MB is always with clinical heterogeneity. To provide patients with more clinically beneficial treatment strategies, there is a pressing need to develop a new prognostic prediction model as a supplement to the prediction outcomes of clinical judgment.

**Materials and Methods:**

Four datasets of mRNA expression and clinical data were downloaded from gene expression omnibus (GEO) database. Differentially expressed genes (DEGs) were identified and functionally enriched among GSE50161, GSE74195, GSE86574. Then we used STRING and Cytoscape to constructed and analyze protein-protein interaction network (PPI) and hub genes. Univariate cox regression analysis was performed to identify overall survival-related hub genes in an unique dataset from GSE85217 as train cohort. Lasso Cox regression model was used to construct the prognostic gene signature. Time-dependent receiver operating characteristic (ROC), Kaplan–Meier curve, univariate and multivariate Cox regression analysis were used to assess the prognostic capacity of the twelve-gene signature. A unique dataset from GSE85217 was downloaded to further validate the results. Finally, we established the nomogram by using the gene signature and validated it with ROC curve. Gene set enrichment analysis (GSEA) was carried out to further investigate its potential molecular mechanism. Besides, the twelve genes expression at the mRNA and protein levels was validated using external database such as Oncomine, cBioportal and HPA, respectively.

**Results:**

A twelve-gene signature comprising *FOXM1, NEK2, CCT2, ACTL6A, EIF4A3, CCND2, ABL1, SYNCRIP, ITGB1, NRXN2, ENAH*, and *UMPS* was established to predict overall survival of medulloblastoma. The ROC curve showed good performance in survival prediction in both the train cohort and the validation cohort. The twelve-gene signature could stratify patients into a high risk and low risk group which had significantly different survival. Univariate and multivariate Cox regression revealed that the twelve-gene signature was an independent prognostic factor in medulloblastoma. Nomogram, which included twelve-gene signatures, was established and showed some clinical benefit.

**Conclusion:**

Our study identified a twelve-gene signature and established a prognostic nomogram that reliably predicts overall survival in medulloblastoma. The above results will help us to better analyze the pathogenesis and treatment of medulloblastoma in the future.

## Introduction

Medulloblastoma (MB), as one of the most common pediatric malignant CNS and brain tumors, has already been classified as a high-risk disease. Patients with MB do not respond well to current treatment and are at increased risk of MB-related death (MacDonald et al., 2014). Epidemiological studies suggested that medulloblastoma is most common between 0 and 9 years of age, and this kind of tumor can occur at any age (Louis et al., 2007). According to the World Health Organization (WHO), there are four major MB histology: classic, desmoplastic/nodular, MB with extensive nodularity, and anaplastic/large-cell (Louis et al., 2007; Polkinghorn and Tarbell, 2007). More recently there are four major molecular subgroups: two associated with pathogenic abnormalities in the wingless pathway (WNT) and sonic hedgehog (SHH) developmental signaling pathways (the WNT and SHH subgroups), and two that are less well molecularly characterized and referred to as Group 3 and Group 4 tumors (Taylor et al., 2012). In addition, Multiple genes expression have been demonstrated to be independent prognostic factors for medulloblastoma. As a member of cyclin-dependent kinases, *CDK4* inhibitor was found to inhibit retinoblastoma protein phosphorylation and cause G1 arrest in patient-derived xenograft models of MB (Cook Sangar et al., 2017). Besides, *CDK6* amplification was among the most common genomic alterations that alter core signaling pathways in SHH-driven MB (Tamayo Orrego et al., 2016). Meanwhile, *CDK6* is also one of the most common recurrent amplifications in Group 3, and Group 4. *CDK6* overexpression had been proved to be an independent prognostic indicator for poor overall survival in patients (Mendrzyk et al., 2005). However, reliable biomarkers that guide MB clinical treatment are few and far between. Therefore, We cryingneed more biomarkers to reduce MB related-mortality and improve MB prognosis. A conventional prognostic assessment tool for MB patients was clinical molecular pathological staging. However, MB is always with clinical heterogeneity. To provide patients with more clinically beneficial treatment strategies, there is a pressing need to develop a new prognostic prediction model as a supplement to the prediction outcomes of clinical judgment.

In the last few years, bioinformatic analysis has been widely used to predict and analyze the functional pathways and genome levels to achieve more precise treatment. Thus, we utilized 3 datasets of MB patients from the Gene Expression Omnibus database (GEO) to identify MB hub genes. Then, we established a gene signature for MB in another GEO dataset and constructed an integrated nomogram by combining various clinicopathological factors, including the twelve-gene signature. Subsequently, the twelve-gene signature was verified in an independent external MB cohort by using unique datasets. Besides, the twelve genes expression in human MB tissues at the mRNA and protein levels was explored using the external database such as Oncomine, the Human Protein Atlas (HPA) databases, and TIMER, respectively.

## Materials and Methods

### Acquisition of Gene Expression and Clinical Data

GEO^1^ is a public database that provides high throughout gene expression data, chips and microarrays (Edgar et al., 2002). Four gene expression datasets (GSE74195, GSE50161, GSE86574, and GSE85217) (Table 1) were downloaded from GEO (de Bont et al., 2008; Griesinger et al., 2013; Amani et al., 2017; Florence et al., 2017). According to the annotation information on the platform, the probes were converted into corresponding gene symbols. After being excluded Patients without survival information, GSE85217 contained 532 MB samples which had clinical information and survival time. The gene expression and clinical information of 323 samples in GSE86217 were used to construct the gene signature as train set. The validation dataset with mRNA expression profile and clinical information used to validate the gene signature was downloaded from the another 109 samples in GSE86217.

**TABLE 1:** Details of the GEO datasets included in this study.

Datasets	References	Platform	Sample size (tumor/control)	Application
GSE74195	de Bont et al., 2008	[HG-U133_Plus_2] Affymetrix Human Genome U133 Plus 2.0 Array	31 (26/5)	Identification of DEGs
GSE50161	Griesinger et al., 2013	[HG-U133_Plus_2] Affymetrix Human Genome U133 Plus 2.0 Array	35 (22/13)	Identification of DEGs
GSE86574	Amani et al., 2017	[HG-U133_Plus_2] Affymetrix Human Genome U133 Plus 2.0 Array	27 (17/10)	Identification of DEGs
GSE85217	Florence et al., 2017	[HuGene-1_1-st] Affymetrix Human Gene 1.1 ST Array [transcript (gene) version]	532 (532/0)	Construction signature and validation

### Identification of Differentially Expressed Gene (DEGs) and Gene Enrichment Analysis

The DEGs was calculated using the “limma” R package (Dalman et al., 2012). After Benjamini–Hochberg (BH) multiple test adjustment, DEGs with absolute log2 fold change (FC) > 1 and ad *P* < 0.05 were considered to be included for subsequent analysis. Enrichment analysis of Gene Ontology (GO) and Kyoto Encyclopedia of Genes and Genomes (KEGG) pathway for DEGs were performed by using the “clusterProfiler” R packag e (Yu et al., 2012).

### PPI Network Construction and Module Analysis

Search tool for the retrieval of interacting genes (STRING)^2^ online database was used to analyze the functional interactions between proteins and provided insights into the pathogenesis and development of diseases (Franceschini et al., 2013). In this study, we used STRING database to construct PPI network. Cytoscape (version 3.4.0), which is an open source bioinformatics software platform for visualizing molecular interaction networks, was performed to draw PPI network (Smoot et al., 2011). Molecular Complex Detection (MCODE) (version 1.4.2), which is an app plug-in Cytoscape, was performed to cluster a given network based on topology to find densely connected regions and identify most significant module (Bandettini et al., 2012). The criteria for selection were as follows: MCODE scores > 5, degree cut-off = 2, node score cut-off = 0.2, Max depth = 100, and k-score = 2.

### Identification of Hub Genes and Survival-Related Hub Genes

The Cytoscape plugin cytoHubba was used to identify hub genes by degree. The hub genes were selected with degrees ≥ 10. The GSE85217 dataset was used to identify hub genes associated with overall survival (OS) by univariate cox regression analysis. Hub genes associated with overall survival with *P* < 0.05 were considered statistically significant and included in subsequent analyses. The univariate analysis was performed using the R packages “survival” and “surveminer” to identify OS-related hub genes (Therneau and Grambsch, 2000).

### Construction of the Gene Signature Model and Validation

Lasso−penalized cox regression analysis was performed to construct the prognostic gene signature. The optimal penalty parameter was estimated by 10-fold cross-validation in the training dataset (Tibshirani, 1997). The prognostic gene signature was presented as risk score = (CoefficientmRNA1 × expression of mRNA1) + (CoefficientmRNA2 × expression of mRNA2) + ⋯ + (CoefficientmRNAn × expression of mRNAn). Talking the median risk score as a cutoff value, 323 patients were divided into high- and low-risk groups. Kaplan Meier (KM) survival curves and time-dependent receiver operational feature (ROC) curve analyses were made to assess the predictive capacity of the model (Heagerty et al., 2002). Besides, the prognostic model was validated in an independent test cohort.

### Prognostic Model Based on Gene Signature as an Independent Predictor for OS and Validation by Using Multiple Databases

Univariate and multivariate cox regression analysis were used to assess whether the prognostic model could be independent of other clinicopathological factors (including age, gender, histology, metastasis staging, molecular subgroup and risk score) (Table 2) for MB patients. Clinical features were selected as an independent variable, and OS was selected as the dependent variable to calculate the hazard ratio (HR) and the 95% confidence interval, two-sided *P*-value. The prognostic genes expression in the gene signature was further validated by using The Oncomine database^3^, TIMER database^4^ (Li et al., 2017) and The Human Protein Atlas database^5^. cBioportal for Cancer Genomics was explored to investigate the genetic alterations of the prognostic genes in the gene signature.

**TABLE 2:** Summary of clinical data.

	Train cohort	Validation cohort
Case included (*n*)	323	109
Age (mean ± SD)	5.4 (± 8.06)	8.6 (± 7.39)
**Overall survival**		
Year (mean ± SD)	3.2 (± 5.13)	2.4 (± 3.31)
Status (alive/dead)	204/119	67/42
**Gender**		
Male	205	73
Female	118	36
**Histology**		
Classic	237	55
Des	40	29
LCA	38	19
MBEN	8	6
**Subgroup**		
WNT	29	6
SHH	86	36
Group3	156	29
Group4	52	38
**Metastasis**		
M0	222	81
M1	101	28

*SD, standard deviation; Des, desmoplastic/nodular; LCA, large-cell anaplastic; MBEN, medulloblastoma with extensive nodularity.*

### Predictive Nomogram Construction and Gene Set Enrichment Analysis

All independent prognostic parameters and corresponding clinical data were included in the construction of a prognostic nomogram via a stepwise Cox regression model to predict 1-, 3-, and 5-year overall survival of medulloblastoma patients in the train set. Then the receiver operating characteristic (ROC) (Heagerty et al., 2002) analysis construction was performed in train set.

### Statistical Analysis

R software version 3.6.2 was used for all statistical analysis. Univariate and multivariate cox regression analyses were performed to evaluate survival situation. The hazard ratio (HR) and 95% confidence interval (CI) were calculated to identify genes related to overall survival. Except as otherwise noted, *P* < 0.05 was considered statistically significant.

## Results

### Identification of DGEs

**FIGURE 1:**
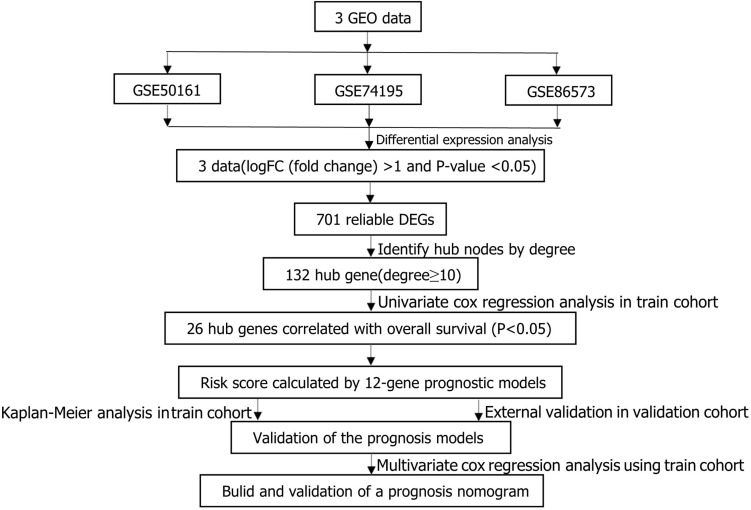
Flowchart presenting the process of establishing gene signature and prognostic nomogram of medulloblastoma in this study.

**FIGURE 2:**
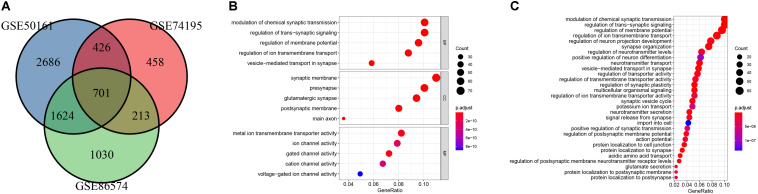
Identification of DEGs and gene enrichment analysis. **(A)** venn diagram of DEGs among the mRNA expression profiling sets GSE74195, GSE50161, and GSE86574. **(B)** Top 5 enriched biological processes (BP), cellular components (CC), molecular functions (MF) of the DEGs. **(C)** KEGG analysis of the DEGs.

The main flow of this study was shown in Figure 1 After standardizing the microarray results, DEGs (1798 in GSE74195, 5437 in GSE50161 and 3568 in GSE86574) were identified. The overlap of the three datasets contains 701 DEGs, as shown in the venn diagram (Figure 2A), consisting of 430 downregulated genes and 271 upregulated genes between MB cancer tissues and normal brain tissues.

### Functional Enrichment and PPI Network Analysis of the DEGs

**FIGURE 3:**
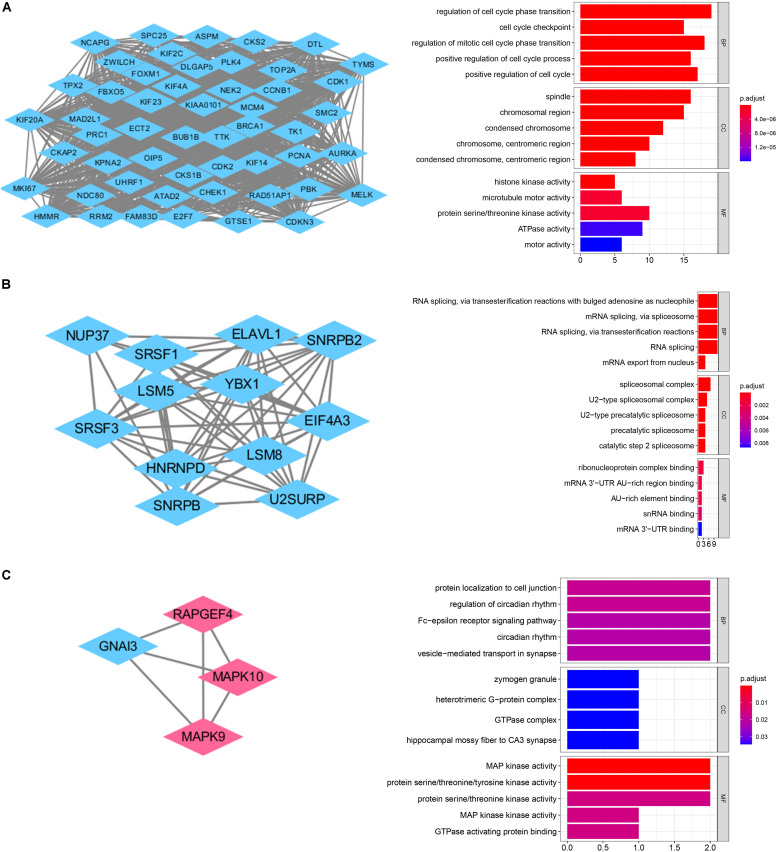
PPI network analysis of the DEGs. **(A)** Clustering module 1 with a score of 48.615 and its top 5 most enriched BP, CC, MF. **(B)** Clustering module 2 with a score of 10.909 and its top 5 most enriched BP, CC, MF. **(C)** Clustering module 3 with a score of 4 and its top 5 most enriched BP, CC, MF.

GO and KEGG pathway enrichment analysis were utilized to analyse the DEGs functions. GO analysis demonstrated that DEGs were found to be significantly enriched in biological processes, such as modulation of chemical synaptic transmission, trans-synaptic signaling, membrane potential, vesicle-mediated transport in synapse, and ion transmembrane transport; cellular components, such as synaptic membrane, glutamatergic synapse, presynapse, postsynaptic membrane, main axon; and molecular functions, such as gated channel activity and metal ion transmembrane transporter activity, ion channel activity, cation channel activity and voltage-gated ion channel activity (Figure 2B). KEGG analysis showed the key pathways correlated with the MB samples: modulation of chemical synaptic transmission, regulation of trans-synaptic signaling and regulation of membrane potential, vesicle-mediated transport in synapse, regulation of ion transmembrane transport (Figure 2C). 132 candidate hub genes (degree ≥ 10) that may play a central role in this network were identified. Module analysis confirmed the important cluster modules in PPI network. The most significant module’ PPI network is with 29 nodes and 371 edges (Figure 3A). Node degree was calculated by the MCC method to obtain hub nodes. The three highest-scoring clustering modules were obtained in Figures 3A–C. GO analysis of Module 1 showed the most significant biological process, molecular function, and cellular component were regulation of cell cycle phase transition, spindle, and histone kinase activity, respectively (Figure 3A). Module 2 with a score of 10.909 was correlated with RNA splicing, spliceosomal complex and ribonucleoprotein complex binding (Figure 3B). Module 3 had a score of 4 and was related to protein localization to cell junction, zymogen granule and MAP kinase activity (Figure 3C).

### Identification of Survival-Related Hub Genes and Establishment of the Twelve-Gene Prognostic Signature

**FIGURE 4:**
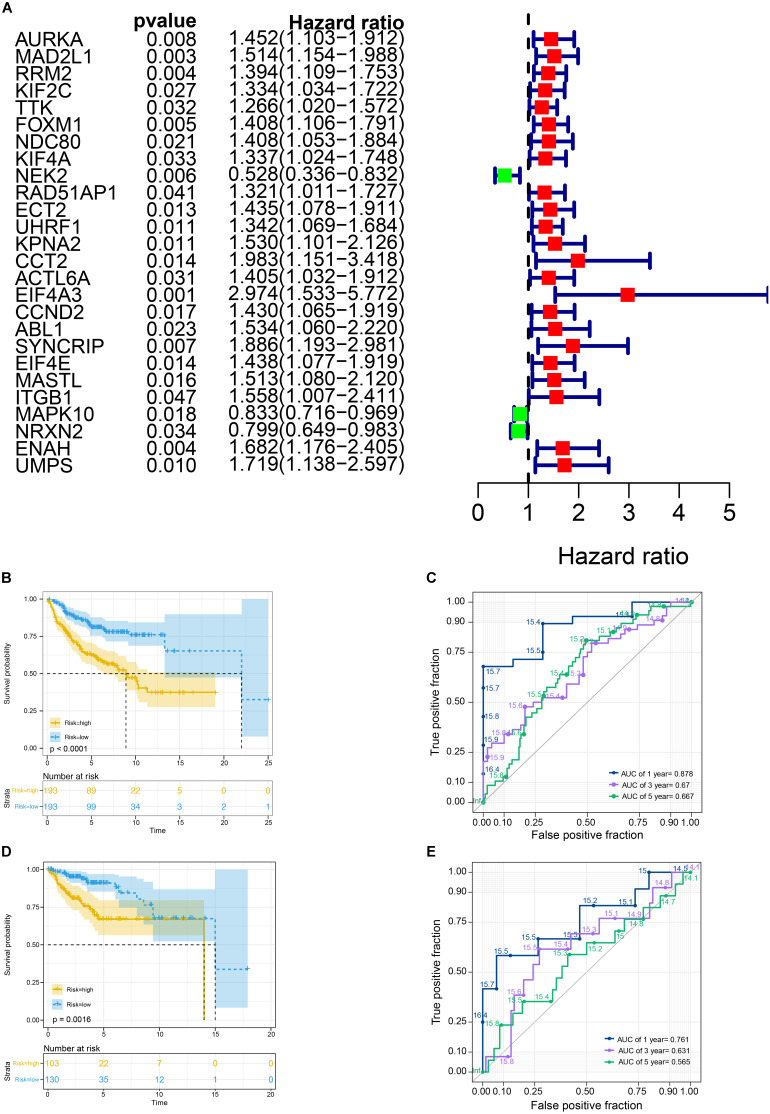
Survival analysis in train cohort and validation cohort. **(A)** Genes associated with OS in train cohort. **(B)** The Kaplan-Meier survival curves of the twelve-gene signature in train cohort. **(C)** The time-dependent ROC curve of the twelve-gene signature in train cohort. **(D)** The Kaplan-Meier survival curves of the twelve-gene signature in validation cohort. **(E)** The time-dependent ROC curve of the twelve-gene signature in validation cohort.

We selected a total of 132 genes as hub genes with degree ≥ 10. Each hub gene was found in ≥ 1 module indicating hub genes that may represent key biological roles in PPI network. 323 patients from the GEO dataset with a follow-up period ≥ 30 days were included in following survival analysis. Based on the univariate cox regression model, a total of 26 hub genes were identified to be significantly associated with overall survival (Figure 4A). Lasso penalized cox analysis identified 12 genes to construct the prognostic model comprising *FOXM1, NEK2, CCT2, ACTL6A, EIF4A3, CCND2, ABL1, SYNCRIP, ITGB1, NRXN2, ENAH, UMPS* (Table 3). The downregulated *NRXN2* was considered as tumor suppressors. The remaining genes were regarded as oncogenes. The risk score = 0.0487 × Expression value of *FOXM1* + (−0.431 × Expression value of *NEK2* + 0.228 × Expression value of *CCT2* + 0.0344 × Expression value of *ACTL6A* + 0.1541 × Expression value of *EIF4A3* + 0.0239 × Expression value of *CCND2* + 0.2674 × Expression value of *ABL1* + 0.193 × Expression value of *SYNCRIP* + 0.3548 × Expression value of *ITGB1* + (−0.0651) × Expression value of *NRNX2* + 0.44 × Expression value of *ENAH* + 0.0066 × Expression value of *UMPS*. Then, we proved findings in the training set by validating the prognostic prediction function of the twelve-gene signature in an unique dataset from GEO.

**TABLE 3:** Functional roles of twelve genes.

Gene symbol	Full name	Function
FOXM1	Forkhead box M1	A transcriptional activator involved in cell proliferation.
NEK2	NIMA related kinase 2	A serine/threonine-protein kinase that is involved in mitotic regulation
CCT2	Chaperonin containing TCP1 subunit 2	A molecular chaperone that is a member of the chaperonin containing TCP1 complex
ACTL6A	Actin like 6A	A family member of actin-related proteins
E1F4A3	Eukaryotic translation initiation factor 4A3	A member of the DEAD box protein family
CCND2	Cyclin D2	A dramatic periodicity in protein abundance through the cell cycle
ABL1	ABL proto-oncogene 1, non-receptor tyrosine kinase	A protein tyrosine kinase involved in a variety of cellular processes
SYNCRIP	Synaptotagmin binding cytoplasmic RNA interacting protein	A member of the cellular heterogeneous nuclear ribonucleoprotein
ITGB1	Integrin subunit beta 1	This gene encodes a beta subunit
NRXN2	Neurexin 2	A member of the neurexin gene family
ENAH	ENAH actin regulator	Members of this gene family are involved in actin-based motility.
UMPS	Uridine monophosphate synthetase	A uridine 5′-monophosphate synthase

### Kaplan–Meier and Time-Dependent ROC Curves of Twelve-Gene Signature

**FIGURE 5:**
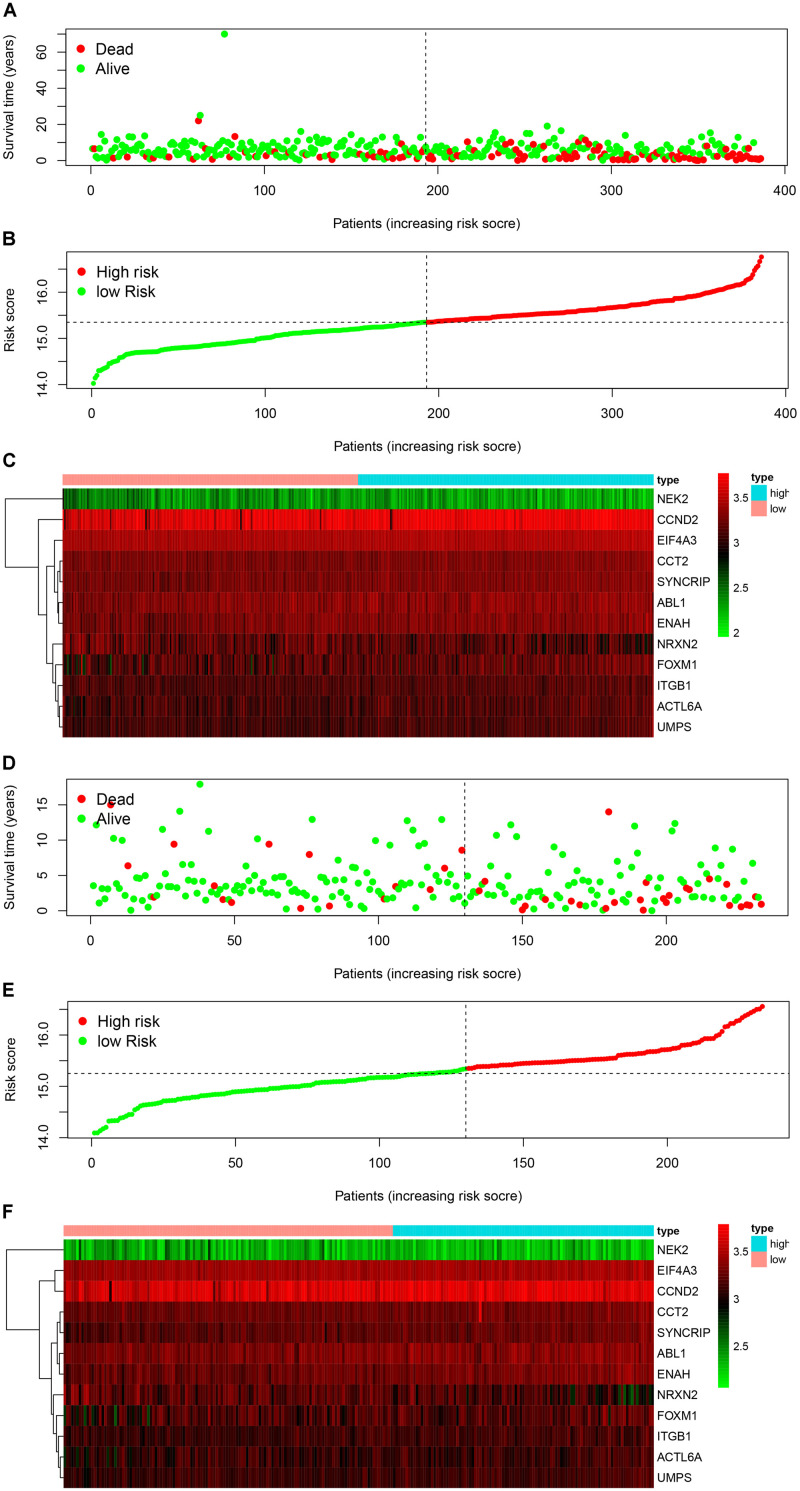
Signature-based risk score in train and validation cohort. **(A–C)** Risk score distribution, survival overview, and heatmap in train cohort. **(D–F)** Risk score distribution, survival overview, and heatmap in validation cohort.

We used the Kaplan-Meier survival curve to compare OS between the two groups divided by the median risk score. In addition, the prognostic prediction capability of the gene signatures was evaluated by using the area under the curve (AUC) of the time-dependent ROC curve. The higher the area under the curve, the better the model performance. the results showed that there was a significant difference on OS between the high- and low- risk groups in train cohort (*P* < 0.0001) (Figure 4B). The AUCs of the twelve-gene signature corresponding to 1-, 3-, and 5- year survival were 0.878, 0.670 and 0.667 (Figure 4C). That means that twelve-gene signature had high sensitivity and specificity in prediction OS. As shown in Figure 4D, the high-risk group prognosis was significantly worse than that of the low-risk group in the independent validation cohort dataset (*P* = 0.0016). The AUCs of the twelve-gene signature model in validation cohort corresponding to 1-, 3-, and 5- year survival were 0.761, 0.631, and 0.565 (Figure 4E), respectively, confirming that the twelve-gene signature has high sensitivity and specificity and can be used as a reliable OS prediction model in MB patients. By comparing the survival status and the twelve-gene expressions of the between high risk and low risk groups, we found that the high-risk group was with poor prognosis (Figures 5A,B). Mean with higher expression of the upregulated identified genes, patients often have a worse prognosis (Figure 5C). In validation cohort, We found the same result the higher the risk score, the worse the prognosis (Figures 5D–F).

### Independent Prognostic Role of the Prognostic Gene Signature

**FIGURE 6:**
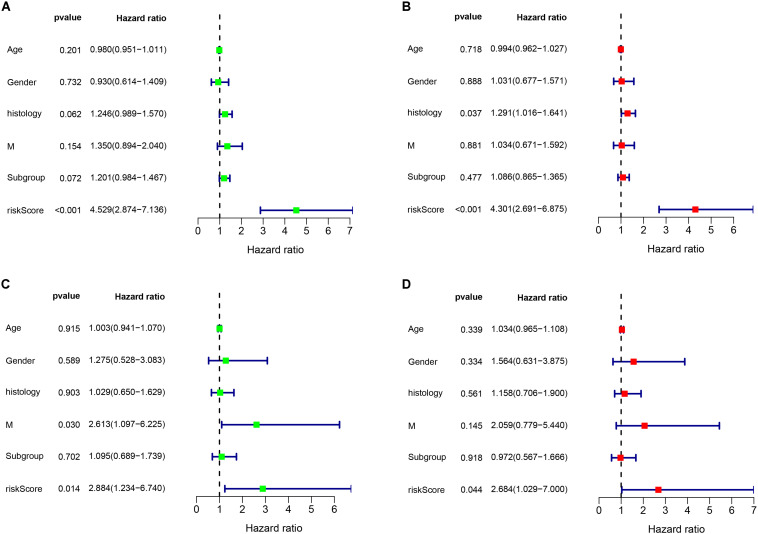
Cox regression analysis of the association between clinical factors and OS. **(A,B)** Univariate and multivariate cox regression analysis in train cohort. **(C,D)** Univariate and multivariate cox regression analysis in validation cohort.

As shown in Figure 6, the risk score can be used as an independent factor in predicting OS (p < 0.05). Among the 323 patients included in train cohort, univariate and multivariate cox regression analysis demonstrated that our prognostic model was an independent prognostic factor for OS, while age, gender, tumor metastasis, molecular subgroup were not associated with OS (Figures 6A,B). Besides, multivariate cox regression analysis showed that histology could be an independent prognostic factor in train cohort (Figure 6B). In test cohort, univariate and multivariate cox regression analysis demonstrated that our prognostic model was also found to be an independent prognostic factor for OS (Figures 6C,D). Meanwhile univariate cox regression showed that tumor metastasis could be an independent prognostic factor in train cohort (Figure 6C). These results further demonstrated that our prognostic model can effectively predict OS of MB patients.

### Validation of the Twelve Gene Expressions

**FIGURE 7:**
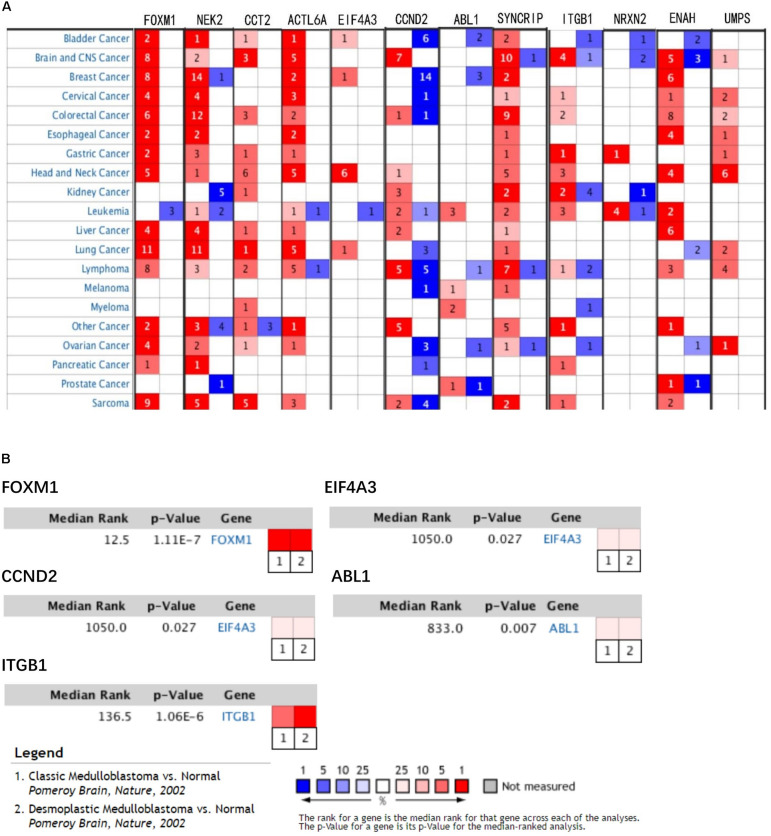
Validation of expression and alteration of the twelve genes (1). **(A)** The expression profiles of the twelve genes in the Oncomine brain and CNS tumor database. **(B)** Meta-analysis of five genes expression in the Oncomine medulloblastoma database. Data of *NEK2, CCT2, ACTL6A, SYNCRIP, NRXN2, ENAH*, and *UMPS* in medulloblastoma were not found in the database.

**FIGURE 8:**
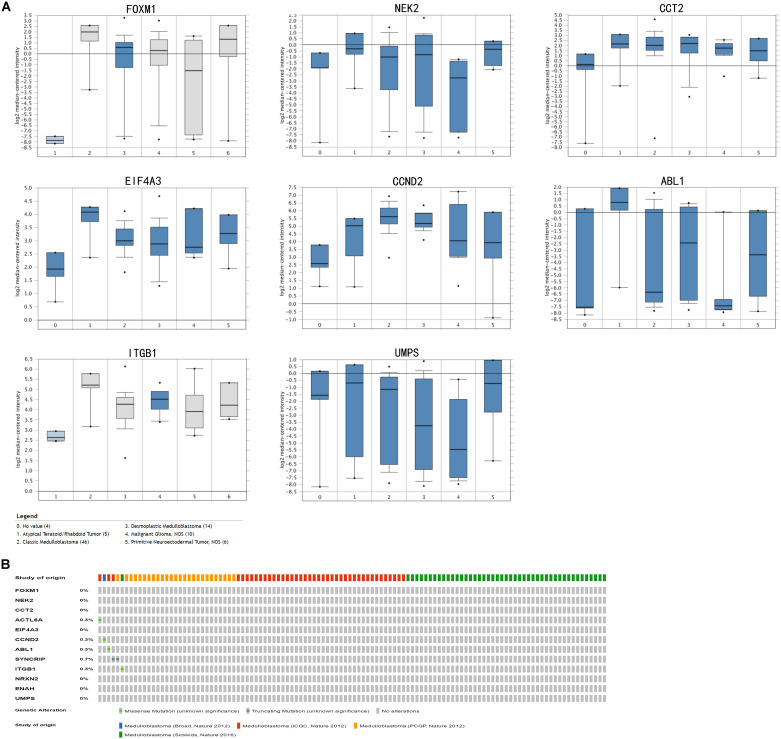
Validation of expression and alteration of the twelve genes (2). **(A)** The expression profiles of the in the Oncomine medulloblastoma database. Data of *ACTL6A, SYNCRIP, NRXN2, ENAH* in medulloblastoma were not found in the database. **(B)** Genetic alterations of the twelve genes in cBioportal medulloblastoma database.

**FIGURE 9:**
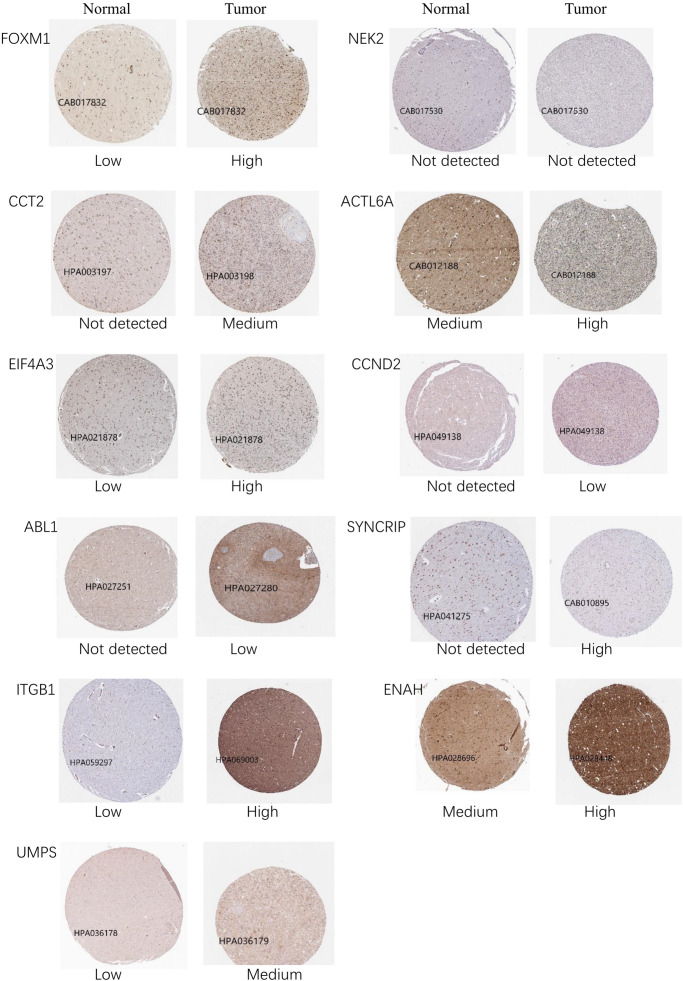
The representative protein expression of the twelve genes in brain tumor and normal brain tissue. Data were from the Human Protein Atlas database. *NRXN2* was not found in the database.

**FIGURE 10:**
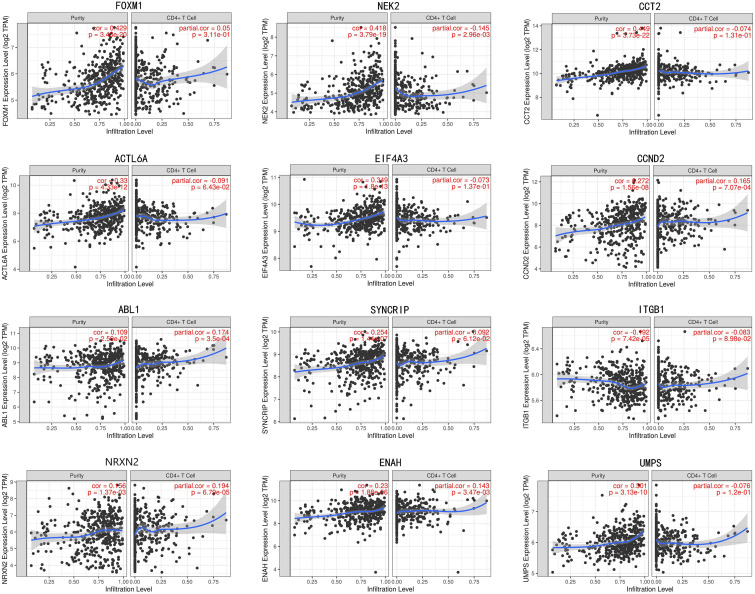
The correlation of twelve genes expression with immune infiltration level in brain tumor.

In train cohort, we found that *FOXM1, NEK2, CCT2, ACTL6A, EIF4A3, CCND2, ABL1, SYNCRIP, ITGB1, ENAH, and UMPS* were overexpressed in MB tissue, while *NRXN2* was underexpressed in MB tissue. Then We tried to validate the twelve genes expression in Oncomine database. Consistent with our results in train cohort, the average expression levels of *FOXM1, NEK2, CCT2, ACTL6A, EIF4A3, CCND2, ABL1, SYNCRIP, ITGB1, ENAH, and UMPS* in CNS and brain tumor tissues were significantly higher than those in normal tissues. However, *NRXN2* expression was significantly lower in in CNS and brain tumor compared to normal tissues which was also consistent with our results (Figure 7A). The results strongly suggested that these twelve genes played an important role in growth of CNS and brain tumor. In order to synthesize multiple research results, we utilized meta-analysis for further confirming the twelve genes expression in MB tissues. In current ococmine meta-analysis, the expression of *FOXM1* and *ITGB1* in MB is highly overexpression, and the conclusion is well supported by all the aforementioned studies (Figure 7B). Besides, *EIF4A3*, *CCND2* and *ABL1* were also expressed in MB tissues in meta-analysis. However, *NEK2*, *CCT2*, *ACTL6A*, *SYNCRIP*, *NRXN2*, *ENAH*, and *UMPS* were not found on the website. With the change of histology, the expression level of *FOXM1, NEK2, CCT2, EIF4A3, CCND2, ABL1, ITGB1*, and *UMPS* were significant difference (except *EIF4A3*, *SYNCRIP*, *NRXN2*, and *ENAH* not included in the database) (Figure 8A). To validate the genetic alterations of the twelve genes in MB tissue, We used 4 MB studies in the cBioportal to investigate. Of the MB patients included in the current study, 1.9% presented with alterations in the twelve genes. *ACTL6A, CCND2, ABL1* and *ITGB1* possessed the missense mutation (0.3%) respectively and truncating mutation in *SYNCRIP* was the most common alteration (0.7%) (Figure 8B). To determine the clinical relevance of these twelve genes expression, HPA clinical specimens were used to analyze the proteins’ expression encoded by these twelve genes (Figure 9). Relative to its expression level in normal brain tissue, *FOXM1*, *ACTL6A*, *EIF4A3*, *ENAH*, and *ITGB1* were strongly positive, while *ABL1*, *CCT2*, *SYNCRIP*, *CCND2*, and *UMPS* were moderately positive in MB tissues. However, *NEK2* and *NRXN2* were not found on the website. In the field of immunology, the abundances of CD4 + T cell were further estimated using the TIMER algorithm. Consistently, the downregulated *FOXM1, NEK2, CCT2, ACTL6A, EIF4A3, CCND2, ABL1, SYNCRIP, ITGB1, NRXN2, ENAH, UMPS* was positively correlated with CD4 + T cell infiltration level (Figure 10). However, our results on *NRXN2* are contrary to the database. Our team speculated that the main reason was that the data from external databases came from CNS and brain tumors, Hence the inconsistent results.

### Building and Validating a Predictive Nomogram

**FIGURE 11:**
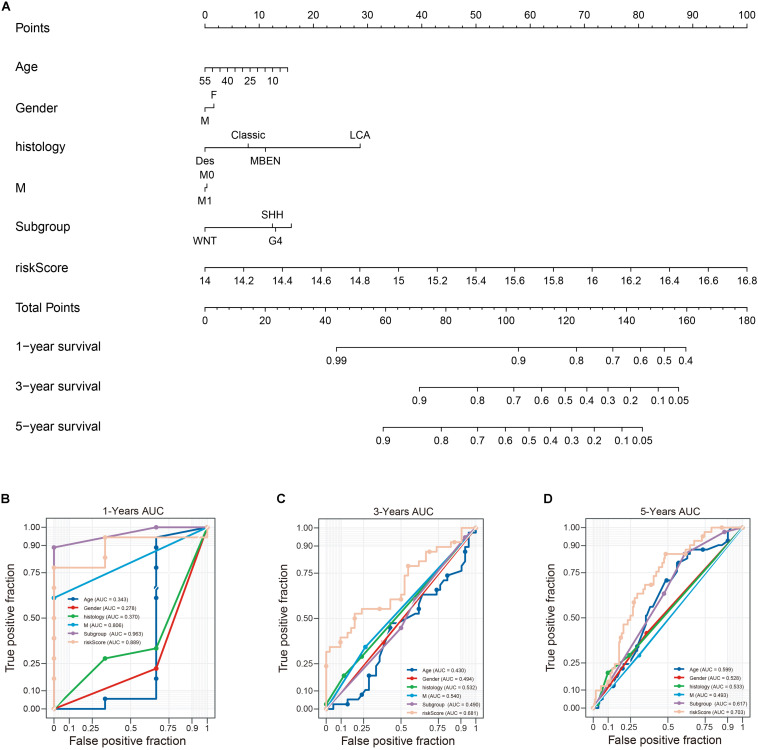
Construction of a nomogram for survival prediction. **(A)** nomogram combing twelve-gene signature and clinical factors, **(B–D)** time-dependent ROC curve analysis of nomogram. Des, desmoplastic/nodular; LCA, large-cell anaplastic; MBEN, medulloblastoma with extensive nodularity.

To establish a clinically applicable way for predicting the survival probability of patients with MB, we developed a nomogram to predict the OS probability in train cohort. All independent prognostic parameters and relevant clinical parameters were included in construction of a prognostic nomogram via a stepwise Cox regression model to predict 1-, 3-, and 5- year overall survival of MB patients in the train cohort (Figure 11A). Time-dependent ROC curve analysis was used to evaluate the prediction accuracy of the integrated nomogram. Although 1- year AUC of the subgroup is the largest, the 1-year AUC of the nomogram was above 0.889 (Figure 11B). Besides, 3 and 5 years of AUCs of the integrated nomogram in Figures 11C,D were the largest suggesting the our nomagram have high predictive accuracy and sensitivity. Besides, we found that the 3 and 5 years AUC of the model is lower than that of 1-year, suggesting that the short-term prediction ability of nomogram may be stronger than the long-term prediction ability.

### Gene Set Enrichment Analysis (GSEA)

**FIGURE 12:**
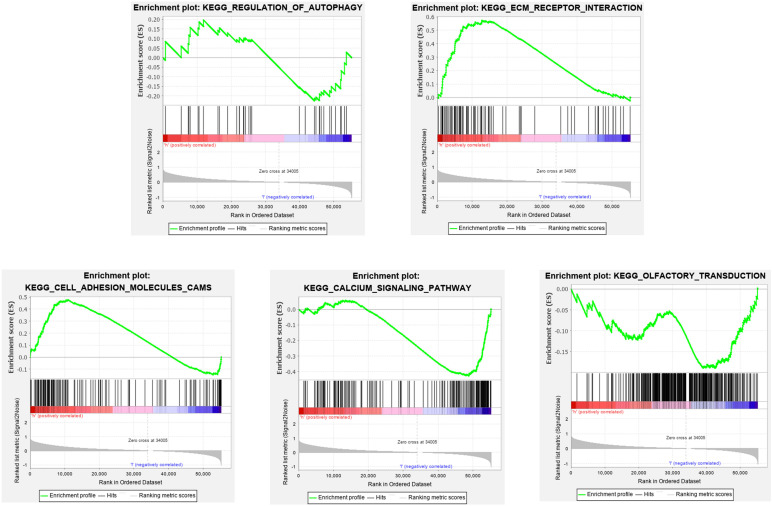
Gene set enrichment analysis (GSEA).

To elucidate the molecular mechanism of the twelve-gene signature, 323 patients from train cohort were divided into high- and low-risk groups. In train cohort, Top 5 KEGG pathways enriched in regulation of autophagy, ecm receptor interaction, cell adhesion molecules cams, calcium signaling pathway and olfactory transduction (Figure 12). The Normalized Enrichment Score (NES) in regulation of autophagy is −0.69112056, Besides FDR q-value in in regulation of autophagy is 0.8572637. In ecm receptor interaction, the NES and FDR q-value were 1.7462078 and 0.05238891, respectively. The NES and FDR *q*-value were 1.5948162 and 0.09338858 respectively in cell adhesion molecules cams. In calcium signaling pathway, the NES and FDR *q*-value were −1.5085816 and 0.22675242, respectively. The NES and FDR *q*-value were −0.8284878 and 0.71028847, respectively, in olfactory transduction. With enrichment score > 0, the left side of the genes corresponding to the peak value of the regulation of autophagy, ecm receptor interaction, cell adhesion molecules cams, calcium signaling pathway are core enrichment genes. With enrichment score < 0, the right side of the genes corresponding to the peak value of olfactory transduction pathway are core enrichment genes. The GSEA results showed the correlation of risk level. Gene sets regulation of autophagy, ecm receptor interaction and cell adhesion molecules cams were enriched in high risk group. Gene sets calcium signaling pathway and olfactory transduction were enriched in low risk group.

## Discussion

Medulloblastoma is the common malignant tumor with poor prognosis in children CNS system. The mechanism of the medulloblastoma development is still controversial. Medulloblastoma is histopathologically divided into four subgroups, and the four subgroups have different mutated genes. With the development of molecular subgroups, gene detection plays an important role in classification and treatment of medulloblastoma. In WNT subgroup, approximately 85–90% of patients harbor somatic activating mutations in exon 3 of *CTNNB1* which lead to constitutively active WNT signaling through stabilization of β-catenin (Waszak et al., 2018). WNT-MB patients lacking somatic *CTNNB1* mutations often carry disease-causing constitutional mutation of the tumor suppressor gene *APC* (Waszak et al., 2018). Several recurrently mutated genes have been identified in WNT subgroup. Most notably, *DDX3X* (in 36% of patients), *SMARCA4* (19%), and *TP53* (14%), as well as clinically actionable mutations in *CSNK2B* (14%), *PIK3CA* (11%), and *EPHA7* (8%) (Northcott et al., 2017).

As another subgroup, genetically most SHH-MB patients have germline or somatic mutations and copy number changes of key genes in the SHH signaling pathway. Assessing the status of *TP53* mutations is important for patient stratification because these mutations are associated with poor prognosis in SHH-MB patients (Kool et al., 2014). It is generally recommended that all patients with SHH-MB should be analyzed for minimal mutations of *PTCH1*, *SUFU*, and *TP53* in tumor and blood (Waszak et al., 2018). Only a DNA-based methylation or expression-based approach can reliably distinguish Group 3 MBs from Group 4 MBs (Cho et al., 2011). The presence of *MYC* or *MYCN* amplification may further increase the stratification of patients in the Group 3 (Shih et al., 2014). Therefore, treatments can be tailored to patients by different biomarker to improve prognosis. Molecular prognostic markers that can be quantified by standardized inspection technique vary with tumor progression and may dynamically reflect the patient’s prognosis. To conquer the hinder of heterogeneity, a group of molecular markers may be more accurate in reflecting MB prognosis than a sole one.

In present study, three mRNA microarray datasets were analyzed to obtain DEGs between MB tissues and normal brain tissues. By using a combined approach of microarray data analysis-bioinformatics tools, the DEGs between MB tissue and normal brain tissue were identified. Then we selected 132 DEGs as hub genes with degrees ≥ 10. Survival analysis revealed 26 hub genes associated with overall survival. identified. Univariate, LASSO, and multivariate cox analysis were used to further narrow the marker range and establish a risk model for predicting MB prognosis. Twelve-gene signature predicting overall survival of MB was established by Lasso cox regression. *NRXN2* was downregulated and identified as protective genes whereas *FOXM1*, *NEK2*, *CCT2*, *ACTL6A*, *EIF4A3*, *CCND2*, *ABL1*, *SYNCRIP*, *ITGB1*, *ENAH*, and *UMPS* were upregulated and associated with poor survival. We evaluated the model performance using the ROC curve of the twelve-gene signature. The results showed that the AUCs of the ROC curves for 1-, 3-, and 5-year survival prediction models were 0.878, 0.670, and 0.667, respectively. That means that the gene signature had high sensitivity and specificity. Besides, We further validate the model in a separate data set.

Our results strongly convinced us that our model can predict the MB patients prognosis better than traditional clinical factors. Finally, we constructed a nomogram that can predict the OS in MB patients. 1-, 3-, and 5- years of AUCs of the integrated nomogram in were the largest suggesting the our nomogram have high predictive accuracy and sensitivity.

To better demonstrate the molecular mechanism underlying MB, we identified 701 DEGs and performed GO and KEGG enrichment analysis for these genes. The results demonstrated that the DEGs were significantly associated with modulation of chemical synaptic transmission, synaptic membrane and gated channel activity. All of these molecular biological processes had been reported in medulloblastoma. A comparison of mouse cerebellar development and medulloblastoma showed that synaptic transmission and other brain-specific neural processes were abnormally developed in medulloblastoma (Liu and Kohane, 2009). Besides, a GO analysis revealed that these DEGs were significantly enriched in gated channel activity. Inhibition of K + channels is an important mechanism by which HO-1 enhances apoptosis resistance of medulloblastoma cells (Al-Owais et al., 2012). Voltage-gated potassium channel also controls mitotic entry and tumor growth in medulloblastoma (Dorand et al., 2016). That is to say, gated channel activity is a worthy target for research on medulloblastoma. Our KEGG enrichment pathway also demonstrated cell and synaptic transmission in medulloblastoma deserved further study to verify.

Seven of the genes in the twelve-gene signature were previously reported to be associated with medulloblastoma. *FOXM1*, a proliferation specific oncogenic transcription factor, is deregulated In a variety of solid tumors (Senfter et al., 2019). *FOXM1* is highly expressed in all medulloblastoma molecular subgroups (Priller et al., 2011). Besides, *FOXM1* expression level significantly correlated with unfavorable clinical outcome in univariate and multivariate analysis (Priller et al., 2011). *NEK2* has been suggested in the regulation of centrosome separation and microtubule organization (Xia et al., 2015). The testing of the *NEK2* as a top candidate showed a strong dependency of medulloblastoma cells on the activity of this enzyme (Boulay et al., 2017). Studies showed that *NEK2*, OTX2 target gene, was knockdown and pharmacological inhibition decreased medulloblastoma cell viability (Boulay et al., 2017). *CCND2*, coding for the cyclin D2 protein, is a cell cycling central regulator. Study showed *CCND2* involved in the sonic hedgehog signaling pathway, were indicted as associated with MB prognosis (Dahlin et al., 2015). Alternative splicing mediated by mutant U1 snRNA activates oncogene *CCND2*, and may be a potential therapeutic biomarker (Suzuki et al., 2019). *ABL1* is a ubiquitously expressed non-receptor tyrosine kinase. *ABL1* has various functions, and cell proliferation can be negatively regulated by nuclear *C-ABL* (Gourlay and Ayscough, 2005). *ABL* contraction mediated by residues G302 and G325. Mutants of these residues, G302V and G325E are associated with medulloblastoma biological process (Epling et al., 2015). *MYC* amplification predict poor prognosis in Group 3 MB. Highly expressed proteins associated with *MYC*-amplified tumors were significantly related to glycolytic metabolic pathways via *SYNCRIPs* (Staal et al., 2015). *ITGB1* is mainly related to the ability of tumor metastasis to promote primary tumor exosmosis, cell adhesion, intravenous injection and tumor growth at metastatic sites (Sheldrake and Patterson, 2014). The reduced expression of *miR-192* was confirmed in the medulloblastoma cells. *MiR-192* decreased cellular anchoring via the repression of *ITGB1* (Yang et al., 2015). *CCT2* is a molecular chaperone that is a member of the chaperonin containing *TCP1* complex. Although *CCT2* has not been reported in medulloblastoma, *CCT2* is significantly enriched in the WNT pathway which is closely related to the growth of medulloblastoma (Yang et al., 2019).

The roles of *ACTL6A*, *EIF4A3*, *ENAH*, and *UMPS* in medulloblastoma had not been reported. However, these genes had also been reported to play a key role in tumors. *ACTL6A* and *P63* interact physically to synergistically control transcriptional programs that promote tumor proliferation and inhibit differentiation (Saladi et al., 2017). *ACTL6A* is vital for embryogenesis and differentiation and is also critical for metastasis in hepatocellular carcinoma (Zeng et al., 2018). *EIF4A3* is an RNA-binding protein that is a core component of the exon junction complex. Besides, *EIF4A3* is overexpressed at the transcriptional level in common malignancies. These results suggested that *EIF4A3* may be a diagnostic marker or therapeutic target for some types of cancer (Lin et al., 2018). *ENAH* gene encoding activation/vasodilation to stimulate the phosphorylated proteins (ENA/VASP) family proteins, involved in cell adhesion and movement required for the assembly of actin filament. Studies suggested that *ENAH* may play a promoting role in the development of gastric cancer and may be a valuable prognostic marker for patients with primary gastric adenocarcinoma (Wang et al., 2017). *BOK* is a key enzyme positive regulator involved in uridine biosynthesis; namely, uridine monophosphate synthetase (*UMPS*). Studies had shown that *BOK* was of certain significance as a biomarker of 5-FU resistance, and has the potential to develop a *BOK* analog for 5-FU resistance tumor sensitization (Srivastava et al., 2019). All the above reports indicated that these four genes had potential in the study of medulloblastoma. Our study was to validate the above genes in order to have more targeted genes in medulloblastoma to improve patients prognosis. The roles of *NRXN2* in cancer development have not yet been elucidated. *NRXNs* is a group of presynaptic single-channel transmembrane proteins that act as synaptic organizers in mammals. The neurexins consist of three genes *NRXN1*, *NRXN*2, and *NRXN3*. Genomic alterations in *NRXN* genes have been identified in a wide variety of neuropsychiatric disorders, including autism spectrum disorders, schizophrenia, intellectual disability (Kasem et al., 2017). The above reports fully proved the significance of *NRXN2* in the nervous system, which may have direct or indirect effects on the growth of medulloblastoma.

Medulloblastoma is a high-grade malignant CNS and brain tumor with a poor prognosis. Recent advances in tumor immunology may provide better treatments for medulloblastoma and reduce its side effects. Our study suggested the downregulated *FOXM1*, *NEK2*, *CCT2*, *ACTL6A*, *EIF4A3*, *CCND2*, *ABL1*, *SYNCRIP*, *ITGB1*, *ENAH*, *UMPS* was positively correlated with CD4 + T cell infiltration level. This means that with the high expression of 11 genes, CD4 + level in patients decreases gradually, greatly reducing the patients anti-tumor ability. In MB mouse models, disruption of *CDK5* expression led to strong tumor rejection mediated by CD4 + T cells, highlighting an important role for *CDK5* in immune checkpoint regulation. Besides, in a mouse model of spontaneous medulloblastoma, targeted *STAT3* destruction of bone marrow cells altered the presence of CD4 + cell (Abad et al., 2014). The above report also showed these twelve genes have the potential to be immune checkpoints in future study.

Contrast to previous bioinformatics studies of medulloblastoma, present study screened different genes as biomarker of medulloblastoma. Then, based on the selection of hub genes, study used the GEO database to select hub genes associated with prognosis to better demonstrate the relationship between genes and prognosis. Meanwhile, our study performed Lasso−penalized cox regression analysis to build the prognostic gene signature. Our nomogram combined with twelve-gene prognostic signature and clinical parameters may enable clinicians to determine the prognosis of individual patients. As far as we knew, twelve-gene prognostic signature and nomograms based on them described here had not been reported before.

However, there was still certain deficiency in our study. First of all, Ethnic factors associated with sequencing samples and some potential prognostic factors may not be included in the model, limiting its predictive power. Secondly, tumor size and resection extent which can predict medulloblastoma prognosis had not been shown in GEO. Next, we will study the medulloblastoma patients treated in our center, include more clinical parameters for analysis, and set additional endpoints to observe different events result.

## Conclusion

The present study identified 701 DEGs and twelve genes regarded as diagnostic biomarkers for medulloblastoma. The mechanism of synaptic transmission and other brain-specific neural processes associated with medulloblastoma growth is currently considered as a promising anticancer strategy. We have reviewed the literature and found that 7 hub genes have been shown to play a role in the pathophysiological process of medulloblastoma. Their insight mechanisms of action and their use in targeted therapies remained to be scientifically investigated. There are still 5 hub genes which were not widely reported. Our present study offered a new perspective and further studies are needed to elucidate the specific functions of these genes in medulloblastoma.

## Data Availability Statement

Publicly available datasets were analyzed in this study. This data can be found here: the Gene Expression Omnibus (https://www.ncbi.nlm.nih.gov/geo/).

## Ethics Statement

Ethical review and approval was not required for the study on human participants in accordance with the local legislation and institutional requirements. Written informed consent from the participants’ legal guardian/next of kin was not required to participate in this study in accordance with the national legislation and the institutional requirements.

## Author Contributions

SZ conceived, designed, analyzed the data, and wrote the manuscript. JW, YC, and FL conceptualized and developed an outline for the manuscript. ZC, XJ, JZ, and QY made a review of the manuscript. All authors contributed to the article and approved the submitted version.

## Conflict of Interest

The authors declare that the research was conducted in the absence of any commercial or financial relationships that could be construed as a potential conflict of interest.

## Footnotes

1 http://www.ncbi.nlm.nih.gov/geo

2 http://string-db.org

3 https://www.oncomine.org/resource/main.html

4 https://cistrome.shinyapps.io/timer

5 http://www.proteinatlas.org
